# Characterization of Volatile Flavor Compounds and Aroma Active Components in Button Mushroom (*Agaricus bisporus*) across Various Cooking Methods

**DOI:** 10.3390/foods13050685

**Published:** 2024-02-23

**Authors:** Limei Xie, Shaoli Guo, Hongting Rao, Bingying Lan, Baodong Zheng, Ningning Zhang

**Affiliations:** 1 College of Food Science, Fujian Agriculture and Forestry University, Fuzhou 350002, China; 2 Fujian Provincial Key Laboratory of Quality Science and Processing Technology in Special Starch, Fuzhou 350002, China

**Keywords:** button mushroom, cooking methods, GC-MS, E-nose, OAV, aroma recombination, aroma omission, aroma active compounds

## Abstract

To investigate the impact of various cooking methods on the volatile aroma compounds of button mushroom, gas chromatography-mass spectrometry (GC-MS) and electronic nose (E-nose) were utilized for aroma analysis. The results indicated that the E-nose was able to effectively distinguish between the samples prepared using different cooking methods. In the raw, steamed, boiled and baked samples, 37, 23, 33 and 35 volatiles were detected, respectively. The roasting process significantly contributed to the production of flavor compounds, giving button mushroom its distinctive flavor. Sixteen differential aromas were identified based on the *p*-value and VIP value. Additionally, the cluster analysis of differential aroma substances revealed a stronger odor similarity between the steamed and raw groups, consistent with the results of the OPLS-DA analysis of overall aroma components. Seven key aromas were identified through OAV analysis and omission experiments. In addition, 1-octen-3-one was identified as the main aroma component of cooked button mushroom. The findings of the study can be valuable for enhancing the flavor of cooked button mushroom.

## 1. Introduction

In recent years, button mushroom (*Agaricus bisporus*) has been widely studied for its flavor, high amino acid score, biological value and essential amino acid index, both in terms of medicinal and economic benefits [1,2,3]. Button mushrooms are typically not consumed raw but instead undergo various cooking methods such as frying, baking, steaming and boiling before being eaten. Although button mushroom is widely used in culinary applications, there is still a scarcity of studies focusing on the alterations in flavor compounds during its culinary preparation.

Different cooking methods can impart distinct effects on the aroma of food due to differences in temperature, heat transfer and heating medium [4]. In their research, Zhou et al. [5] explored the impact of boiling, steaming, microwaving, deep-frying and pressure cooking on the volatile constituents of shiitake mushrooms and found that pressure cooking and deep-frying produced more aroma active compounds. Similarly, Cheng et al. [6] observed that gas-frying treatments significantly contributed to the creation of flavorful substances, imparting a unique flavor to Tibetan pork. Aroma is a fundamental characteristic of food, and the cooking operation primarily affects the overall aroma of food through mechanisms such as the Maillard reaction [7], lipid oxidation, amino acid degradation [8] and their combined reactions [9]. As of now, approximately 80 volatile compounds, such as aldehydes, ketones, alcohols, acids, terpenes, esters and heterocyclic compounds, have been identified in raw button mushroom [10]. Although button mushroom does not have a strong aroma on its own, the process of cooking can lead to Strecker degradation and Maillard reactions, generating a variety of volatile and non-volatile intermediates, which contribute to the development of a delicious mushroom flavor [11,12]. However, there is little literature that systematically investigates the changes in the flavor of button mushroom during steaming, boiling and roasting.

GC-MS is the most widely used technique for detecting flavor substances, while the E-nose is an instrument comprising a series of electronic chemical sensors with partial specificity. Recently, E-nose and GC-MS methods have been widely used to analyze the volatile components of various food products to identify different species, qualities and processing and storage conditions, such as *Pleurotus eryngii* [13], skim milk [14], low-salt fermented sour fish [15], wine [16], fruits [17] and vegetables [18]. In addition, the E-nose sensor accurately responded to eight different dried commercial mushrooms, indicating its potential as a tool for identifying edible mushrooms [19]. Simultaneously, E-nose has been utilized to distinguish between various cultivated fresh mushrooms, such as *Flammulina velutipes*, *Agaricus bisporus*, *Lentinus edodes*, *Pleurotus eryngii*, *Grifola frondosa* and *Hypsizygus marmoreus* [20]. Gómez et al. utilized an E-nose, GC-MS technique and sensory panel method to analyze the odor of the freeze-dried powder of cultivated *Agaricus bisporus*. The results indicated that the E-nose has analytical utility and can be employed for assessing different types of freeze-dried *Agaricus bisporus* powder [21]. These studies showed that E-noses are highly sensitive in detecting a wide range of food products, and the aroma results obtained through both techniques are strongly correlated. Given that button mushroom is a widely consumed edible mushroom, it undergoes flavor changes during cooking. Consequently, it is essential to comprehend the impact of various cooking methods on the flavor of button mushroom.

Accordingly, the present study selected button mushroom treated with different cooking methods and analyzed their aroma characteristics using E-nose and GC-MS techniques. This study aimed to enrich the existing research on the effects of cooking methods on the flavor and aroma of edible mushrooms, providing additional insights into how cooking affects the aroma of food.

## 2. Materials and Methods

### 2.1. Chemicals

For identification and quantitation of aroma compounds, the following authentic flavor standards were purchased from Macklin Biochemical Technology Co., Ltd., Shanghai, China: methyl valerate (99%), isovaleraldehyde (98%), furfuryl mercaptan (98%), heptanal (97%), hexanal (99%) and 2-butylfuran (98%). Benzyl alcohol (AR, 99%), 1-octen-3-one (98%), 1-octen-3-ol (98%), methyl palmitate (AR, 97%) and benzoic acid (99%) were purchased from Yuanye Bio-Technology Co., Ltd., Shanghai, China. Phenylacetaldehyde (95%) and benzaldehyde (AR, ≥98.5%) were obtained from Sinopharm Chemical Reagent Co., Ltd., Beijing, China. The internal standard, 1-decanol (GC, ≥99.5%), was purchased from Aladdin Biochemical Technology Co., Ltd., Shanghai, China.

### 2.2. Button Mushroom Samples and Cooking Methods

Button mushrooms were purchased from the local Yonghui supermarket (Fuzhou, China). Fresh button mushrooms with non-rotting, non-moldy bodies were carefully selected and washed under running water to remove any loose growth medium from the cap. The washing process lasted for 5–10 s to avoid excessive water retention. The button mushrooms were rinsed and placed in a steel strainer metal bowl to drain well, and excess water was absorbed with a paper towel before removing their stalks and discarding them. Using a slicer, the caps were sliced longitudinally into thin slices, approximately 0.5 cm thick. The first and last slices were discarded due to uneven cooking compared to the other caps. All the sliced mushrooms were randomly divided into four groups, one of which was raw, and the rest were cooked using three methods (steaming, boiling and baking).

We chose cooking conditions according to the methods of Sun et al. [22], Lee et al. [23] and Irene et al. [24] with slight modifications, and the specific cooking treatment conditions were as follows:

1. Steamed: Room temperature water was added at a 1:20 (g/mL) ratio in a steamer. The water was heated to boiling, and the mushroom slices were placed on the steamer rack in the steamer and steamed for 7 min.
2. Boiled: Room temperature water was added at a ratio of 1:20 (g/mL) in a pan and heated on an induction cooker (Guangdong Midea Life Electric Appliance Manu-facturing Co., Foshan, China) until the water boiled. Then, the sliced mushrooms were poured into the pan and were cooked for 7 min.
3. Baked: The oven (Guangdong Midea Life Electric Appliance Manufacturing Co., Foshan, China) was preheated to 163 °C, and the mushroom slices were baked in the 163 °C oven for about 4 min on each side, totaling 8 min.

The cooking time was optimized through systematic experiments based on sensory evaluation and time efficiency. Initial tests covered baking durations of 5, 7, 10 and 15 min, followed by trials at intervals of 7, 8, 9 and 10 min. An 8 min baking time was found to offer superior sensory scores and eliminate raw mushroom flavor. Similar methodologies were applied in determining steaming and boiling times.

After cooking, a portion of all 4 samples (raw, boiled, steamed and baked mushroom) was used for subsequent experiments.

### 2.3. E-Nose Measurement

The flavor characteristics of button mushroom were analyzed according to the method of Guo et al. [25] with minor modifications. Button mushroom samples (2.00 g) were placed in a 20 mL headspace bottle at room temperature for 45 min. The E-nose (PEN3 Airsense, Schwerin, Germany) contains 10 metal oxide gas sensors: W1C (aromatic), W5S (nitrogen oxides), W3C (ammonia and aromatic), W6S (hydrogen), W5C (alkanes and aromatic), W1S (short-chain alkanes), W1W (inorganic sulfur), W2S (alcohols, ethers, aldehydes and ketones), W2W (organic sulfur) and W3S (long-chain alkanes). The E-nose test conditions were as follows: sensor cleaning time of 120 s, injection preparation waiting time of 5 s; sampling time of 120 s, injection flow rate of 400 mL/min and operating ambient temperature of 26 °C. The signal from 81 s to 83 s of the smoothed data was utilized as the time point for the E-nose analysis. Each sample was measured in triplicate, and the average value was used for further analysis.

### 2.4. Headspace Solid-Phase Micro-Extraction (HS-SPME) of Aroma Compounds

By referring to previous studies with some modifications, we employed HS-SPME method to determine volatile compounds in button mushroom [26]. A total of 4 g of samples was homogenized with 10 mL of distilled water, and 5 mL of slurry was mixed with 10 µL (165.94 µg/mL) of 1-decanol internal standard solution before being sealed in a 50 mL vial using a headspace jaw cover with spacer. Nest, the samples were shaken in a 60 °C water bath for 30 min. To extract volatile compounds from button mushroom, a 75 µm divinylbenzene/carboxen/polydimethylsiloxane (DVB/CAR/PDMS, 75 µm) (Agilent Technologies Inc., Santa Clara, CA, USA) fiber was used and preconditioned at 250 °C for 30 min prior to analysis. The aged HS-SPME fiber was exposed to the sample for 40 min at 60 °C to collect the analytes. Afterward, the fiber was inserted into the injection port of the GC-MS system for thermal desorption.

### 2.5. GC-MS Analysis

GC-MS analysis was performed on the Agilent 8860/5977B GC-MS instrument (Agilent Technologies Inc., Santa Clara, CA, USA). Analytes were separated on a DB-5ms capillary column (30 m × 0.25 mm × 0.25 μm) from Agilent Technologies Inc., Santa Clara, CA, USA, in nonsplit mode. The heating-up procedure was as follows: held at 35 °C for 5 min, raised to 100 °C at 5 °C/min, held for 1 min, raised to 230 °C at 10 °C/min and held for 10 min. The total time of the procedure was 42 min. The carrier gas was helium (purity not less than 99.999%) at a constant flow rate of 1.4 mL/min. Mass spectra were acquired in an electron impact mode. MS was taken at 70 eV ionization energy in the 35–550 amu mass range, with a solvent delay of 1.00 min. The ion source temperature was 230 °C, with the quadrupole temperature set to 150 °C. All experiments were performed in triplicate.

Compounds with a match percentage greater than 85% were selected for confirmation based on the NIST standard mass spectrometry data. Through comparison with the peak area of the internal standard, the content of the aroma substance can be quantitatively calculated in µg/g using the following formula: Aroma substance content = (peak area of aroma substance × content of internal standard)/peak area of internal standard.

### 2.6. Calculation of Odor Activity Values (OAVs)

The odor activity value (OAV) was employed to evaluate the contribution of each volatile compound to the overall aroma, thereby enabling a better understanding of the significance of individual aroma substances in the overall aroma profile. The OAV was calculated as the ratio of the semiquantitative analysis result of a single volatile compound to the perception threshold of the substance in water, expressed as OAV = aroma component content/odor threshold. Thresholds and odorant descriptions for volatile compounds were obtained from http://www.odour.org.uk (accessed on 19 February 2024)and the *Compilations of Odour Threshold Values in Air, Water and Other Media*. In general, compounds with OAV greater than or equal to 1 contribute significantly to the overall aroma, while compounds with OAV less than 1 contribute less [27].

### 2.7. Aroma Recombination and Omission Experiments

To further validate the key aroma active compounds of button mushroom, the aroma was reconstituted and compared with the original button mushroom. Aroma reorganization and omission experiments were performed with appropriate modifications based on the methods of Liu et al. [28] and Fan et al. [29]. In summary, the reconstitution experimental model comprised a standard aroma substance with an OAV greater than 1 and ultrapure water, with all compounds present in natural amounts. Further omission experiments were conducted based on the results of the aroma reorganization experiments. By removing a compound from the recombination model, an omission model was obtained. We could then use the E-nose to evaluate the distinctions between each omission model and the recombination model.

### 2.8. Statistical Analysis

Every experiment was performed in triplicate, and results were expressed as the mean value ± standard deviation (SD) of three replicates. The estimation of differences between means was conducted using SPSS 24.0 (SPSS Inc., Chicago, IL, USA) for one-way ANOVA (*p* < 0.05). Radar map was drawn through Origin 2023 (OriginLab Co., Northampton, MA, USA). Clustered heat map analysis was performed using https://www.omicstudio.cn/tool (accessed on 19 February 2024). Simca 14.1 (Umetrics AB, Umea, Vasterbotten, Sweden) was used for OPLS-DA (the scaling type was Par) analyses. Additionally, we employed supervised regression modeling using OPLS-DA to obtain variable importance in projection (VIP). Differential aroma components were screened at *p* < 0.05, VIP > 1.

## 3. Results and Discussion

### 3.1. E-Nose Analysis

**Figure 1:**
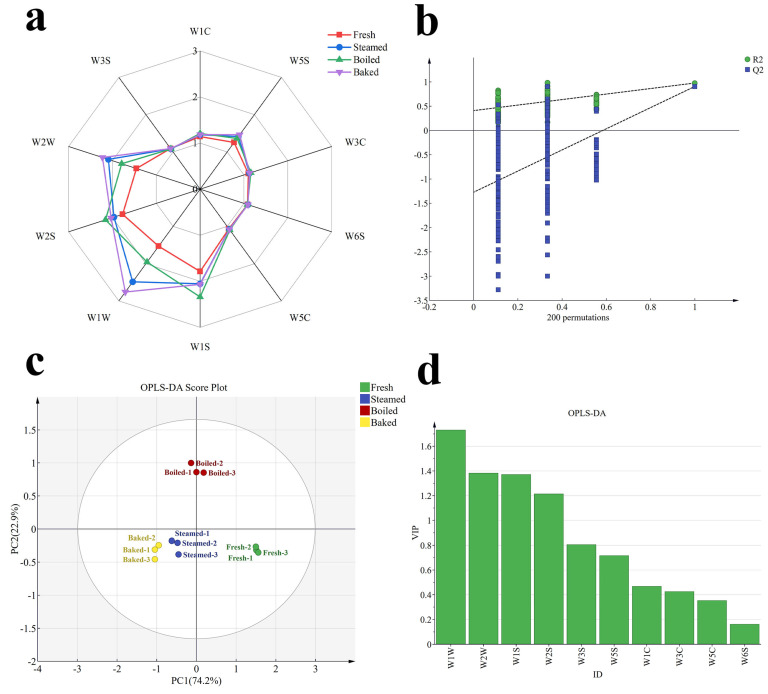
E-nose analysis of button mushroom with different cooking methods. (**a**) E-nose radar diagram. (**b**) Two hundred permutation test chart of OPLS-DA model. (**c**) OPLS-DA analysis of Enose. (**d**) VIP values of OPLS-DA model.

The E-nose is an electronic chemical sensing system that simulates the human sense of smell to rapidly analyze and detect complex volatile components in food. It offers the advantages of accuracy, sensitivity and objectivity [19]. From Figure 1a, the response values of the 10 sensors to the volatile components of button mushroom with different cooking methods were observed to be different. The samples exhibited the strongest response values at sensors W1W and W1S, followed by W2W and W2S, which suggested that sulfides, short-chain alkanes, alcohols, ethers, aldehydes and ketones were the primary volatiles of button mushroom. Meanwhile, high levels of aldehydes and alcohols were found in the roasted group in the GC-MS test. This outcome could be linked to the Maillard reaction, where polysaccharides and proteins in button mushroom undergo chemical changes at high temperatures, resulting in the generation of numerous aromatic compounds during the roasting process [30].

To delve deeper into the impact of distinct cooking methods on the odor of button mushroom, an OPLS-DA model was constructed for analysis, with the findings depicted in Figure 1b–d. According to the analysis results in Figure 1c, PC_1_ and PC_2_ explained 74.2% and 22.9% of the variance, respectively, contributing a total of 97.1% of the variance. This indicated that these two principal components could effectively reflect most of the characteristics of the volatile odors of the samples. The OPLS-DA analysis demonstrated an independent variable fit index (R^2^X) of 0.999, a dependent variable fit index (R^2^Y) of 0.882 and a model prediction index (Q^2^) of 0.584. Both R^2^ and Q^2^ exceeding 0.5 indicated that the model fit was acceptable [31]. Figure 1b indicates that, after 200 permutation tests, the intersection of the Q^2^ regression line with the vertical axis was less than zero, which suggested that there was no overfitting of the model, and the model was valid. Therefore, the results were deemed useful for the discriminant analysis of the cooking style of button mushroom. In Figure 1c, the raw group was depicted as distributed in the positive semi-axis of PC_1_, while the cooking-treated samples were overall distributed in the negative semi-axis of PC_1_. This suggested that the E-nose technique effectively distinguished between raw and cooked samples, highlighting a significant alteration in the overall distribution of volatile aromas of button mushroom due to the cooking treatment. Furthermore, button mushroom successfully differentiated between the three cooking styles on the score scatterplot. The roasted and steamed groups were evenly distributed on the negative half-axis of PC_2_, with a small distance between them. This could mean that in terms of overall flavor, the roasted and steamed samples had some similarity in some components. Conversely, the boiled samples were uniformly distributed in the positive semi-axis of PC_2_, indicating a significant difference in flavors compared to the other two groups. The reason may be that the exposure of button mushroom to drying at high temperatures during roasting resulted in the rapid evaporation of water and significant shrinkage of the mushroom surface, which may have allowed better retention and release of volatile components. On the other hand, the boiling treatment may have led to the partial dissolution of flavor substances into water, resulting in losses [5,32].

In addition, Figure 1d displays the VIP values based on the OPLS-DA model, which measured the response value of each sensor and characterized the degree of variable contribution. The higher the VIP value (VIP > 1), the greater the amount of substances in the flavor of button mushroom. All four sensors, W1W, W2W, W1S and W2S, had VIP values greater than 1, suggesting that these four types of odors were stronger in button mushroom, and it was thought that these sensors may have contributed more to the OPLS-DA model. In the meantime, the W1W and W2W sensors showed higher sensitivity to sulfide and contributed the most in this test, which indicated the presence of sulfide in button mushroom. In addition, this was verified by the GC-MS analysis results, where higher levels of benzyl mercaptan were detected in both roasted and boiled samples. Benzyl mercaptan is a common sulfide with a distinctive odor, often described as similar to that of onions or garlic [10]. Therefore these findings were crucial for understanding how the odor characteristics of button mushroom relate to its culinary treatments.

### 3.2. Characterization of Volatile Compound Species

The E-nose differs from commonly used methods for identifying flavor substances, such as GC-MS. It provides an overall flavor map rather than a map of individual components, and thus, it cannot qualify or quantify a single flavor component [33]. Table 1 displays the results of the analysis of the aroma components of button mushroom using GC-MS. A total of 73 compounds were detected, including 12 alcohols, 20 esters, 12 aldehydes, 5 acids, 9 ketones and 15 others.

In order to further grasp the effect of cooking treatments on aldehydes, alcohols, ketones, acids and esters in button mushroom, their types and contents are summarized in Table 2. The results show that 37, 23, 33 and 35 volatile components were identified in the four groups of samples, which indicates that different cooking treatments had a significant effect on the types of volatile components of button mushroom. In particular, the steaming treatment led to a significant reduction in the variety of volatiles. Meanwhile, the grilling treatment helped to retain the volatile flavor compounds in button mushroom.

Based on the findings in Table 1, the primary volatile components of fresh button mushroom contained isovaleraldehyde, benzoic acid, palmitic acid, benzyl alcohol, 3-octanol and others. However, recent studies have indicated that the major volatile constituents of fresh button mushroom were eight-carbon volatiles, such as 1-octen-3-ol, 3-octanol, 2-octen-1-ol, 1-octanol and 3-octanone. These compounds have been found to vary in types and quantities [34]. For example, Maga’s [35] study demonstrated that the content of 1-octen-3-ol and 1-octen-3-one increased with storage time, where 1-octen-3-ol was associated with the aroma of mushrooms, while 1-octen-3-one was not. Çağlarırmak conducted an enzymatic reaction to extract volatile compounds and subsequently measured them. The findings revealed that the volatile components in fresh button mushroom predominantly comprised C18 or C16 compounds, such as octadecanoic acid, hexadecanoic acid derivatives and other significant volatiles [36]. The study by Pei et al. [37] investigated the impact of two drying methods on the volatile constituents of button mushroom. Their findings revealed that fresh mushrooms contained high concentrations of C8 compounds (e.g., 1-octen-3-ol, 3-octanol, 2-octen-1-ol and 3-octanone), as well as benzyl alcohol, benzaldehyde and aromatic esters. Therefore, the main volatile components of fresh button mushroom found in the present study were slightly different from the previous studies, which might be due to the differences in freshness and extraction methods. Furthermore, future experiments should be conducted to investigate the specific effects of freshness and extraction methods on the flavor of button mushroom, with the aim of further elucidating its primary flavor substances.

Alcohols, commonly characterized by a sweet, fruity and vegetal aroma, are an essential component of the flavor profile of edible mushrooms [38]. According to Table 2, seven alcohols were detected in the raw group. After the cooking treatment, the number of alcohol substances decreased in all groups. Notably, the roasted group exhibited the highest concentration of alcohols at 54.81 µg/g, suggesting that the roasting process contributed to the release of more alcohols. This may be due to the fact that the grilling treatment increased the contact area of hot air with the lipids in button mushroom, which induced peroxidation reactions to produce more alcohols [39]. Benzyl alcohol is an important aromatic alcohol that imparts floral and rosy flavor to food [40]. According to Table 1, benzyl alcohol was the most abundant alcohol compound. Compared to the raw group, boiling and roasting treatments led to a significant increase in benzyl alcohol content, with the highest level of 47.56 µg/g in the roasting group. Conversely, the steamed group exhibited a notably lower benzyl alcohol content, suggesting a loss of this compound due to the steaming treatment. Furthermore, we observed that the abundance of the eight-carbon volatiles 3-octanol and 1-octen-3-ol was lower in the cooked group, which could be attributed to the instability of these volatiles under high-temperature conditions, leading to their degradation and loss [41]. These findings were consistent with previous studies by Du et al. [42], Ashmore et al. [43] and Huang et al. [44]. In summary, roasting facilitated the release of alcohols from button mushroom, thereby enhancing the overall flavor profile.

Esters, the third most abundant volatile organic compounds in edible mushrooms, are primarily formed through the esterification of acids with alcohols [45]. These compounds usually present sweet and fruity and significantly influence the overall flavor profile of food due to their low odor threshold [46]. As shown in Table 2, cooking treatments significantly reduced the number of ester compounds, causing an adverse impact on the ester compounds. The ester compounds mainly include ethyl acetate, bis(2-ethylhexyl) phthalate, L-ascorbyl dipalmitate, heptadecanoic acid, heptadecyl esterr, etc., which may be derived from the oxidation reaction of aldehydes [27]. Specifically, ethyl acetate, known for its fruity flavor profile, exhibited a significant decrease following cooking. In summary, the cooking treatments induced changes in ester compounds, potentially impacting the flavor profile of button mushroom.

Aldehydes are crucial in shaping the overall odor of food due to their low odor threshold and potent odor properties [44]. According to Table 2, only the steamed group showed a significant decrease (*p* < 0.05) in aldehydes compared to the fresh group, while both boiling and baking treatments resulted in a significant increase in aldehydes. In particular, the total aldehyde content in fresh samples increased from 10.73 µg/g to 44.76 µg/g after the roasting treatment. According to Table 1, the main aldehydes found in button mushroom were benzaldehyde, isovaleraldehyde, furfural and heptanal. Benzaldehyde, known for its sweet almond flavor, was a product of benzoic acid and had been demonstrated to contribute to the flavor of mushrooms [47]. Compared to the raw group, the boiled and roasted groups showed a significant increase in benzaldehyde content, especially in the roasted group. This could be attributed to the degradation of benzoic acid to benzaldehyde in fresh samples during roasting [48]. Furthermore, the baking treatment resulted in a notably higher isovaleraldehyde content compared to the other groups. This increase could be attributed to the high temperature and dry environment of the oven, which induced amino acid catabolism in the samples, leading to the Strecker degradation reaction of leucine, resulting in the production of isovaleraldehyde in substantial quantities [49]. Isovaleraldehyde, known for its low-threshold flavor, with apple aroma and malt odor [50], could significantly enhance the overall flavor of the food and was popular among consumers. Furthermore, Schmidberger et al. [51] identified isovaleraldehyde as an aroma active compound in truffles. Similarly, Vahdatzadeh and Splivallo [52] demonstrated that leucine, isoleucine, phenylalanine and methionine were important flavor precursors in edible mushrooms through ^13^C-labeling experiments. They found that benzaldehyde was a product of phenylalanine catabolism, while isovaleraldehyde was a catabolism product of leucine. In addition, furfural, which had fatty and almond flavors, was found to be the highest in the boiled samples (4.06 µg/g). All three cooking methods significantly increased the content of heptanal compared to the raw group, indicating that cooking treatments contributed to the production of heptanal. In conclusion, aldehydes played a crucial role in the odor characteristics of button mushroom. The significant production of aldehydes during cooking may contribute to the overall flavor of button mushroom, whereas grilling treatments may contribute to the release of aldehydes from button mushroom.

According to Table 2, cooking treatments significantly altered the content of acids. Boiling significantly increased the content of acids, while steaming and roasting significantly decreased the content of acids. Most of the acids had high odor thresholds and were associated with unpleasant odors, so they had relatively little effect on the aroma profile of button mushroom [53].

After boiling, the highest ketone content was observed, with 3-octanone and 1-octen-3-one being the most abundant ketones produced. 3-octanone presented an earthy and mushroomy flavor, while 1-octen-3-one offered a strong mushroom aroma, enhancing the fragrance of button mushroom [54]. Ketones, resulting from amino acid degradation and fatty acid oxidation, primarily contributed a plant fragrance smell to button mushroom and played a role in the Maillard reaction [55]. Consequently, boiling was more conducive to the release of ketones.

Other compounds such as phenols, terpenes and alkanes also influenced the flavor of button mushroom. Particularly, elevated levels of benzyl mercaptan were observed in the boiled and roasted groups, aligning with the response values of the W1W and W2W sensors in the E-nose analysis. Furthermore, 2-methylpyrazine and cycloheptatriene were detected in the baked group, suggesting that the high-temperature convective air of the oven prompted the production of additional heterocyclic molecules in button mushroom.

Based on the above discussion, it seemed that the two methods, boiling and baking, were more conducive to the release of flavor substances from button mushroom. However, in summary, the most abundant types and contents of flavor substances were released from button mushroom after the baking treatment.

### 3.3. Differential Aroma Analysis

**Figure 2:**
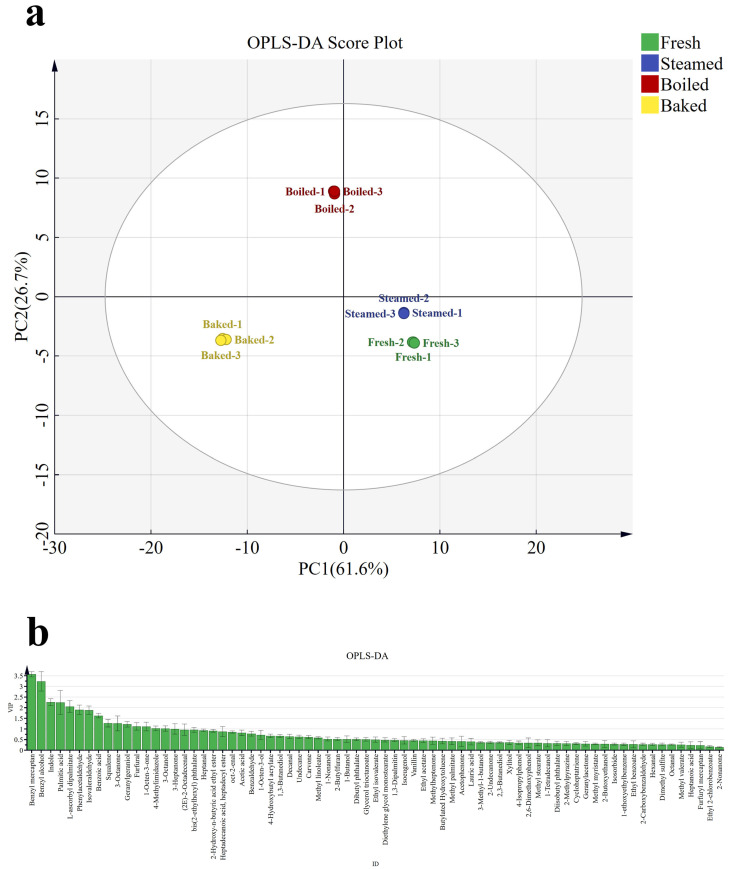
OPLS–DA analysis of volatile substances. (**a**) Plot of OPLS–DA scores of volatile flavor components. (**b**) VIP values of volatile flavor components.

The species characterization of volatiles clarified that cooking treatments had a significant effect on the aroma of button mushroom. To effectively differentiate the flavor of button mushroom under different cooking methods, we performed an OPLS-DA analysis with 73 aroma components as dependent variables and different cooking methods as independent variables (see Figure 2). From Figure 2a, the steamed group was positioned on the positive semi-axis of PC_1_, closely resembling the raw group, indicating a similarity in volatile components between the steamed and raw groups. In addition, the roasted and boiled groups were uniformly distributed on the negative half-axis of PC_1_, which implied a significant difference in flavor compared to the other samples, probably due to the sulfides detected in the roasted and boiled samples. The VIP values are shown in Figure 2b in accordance with the OPLS-DA model. Based on the criteria of VIP > 1 and *p* < 0.05, 16 differential aroma compounds (as shown in bold in Table 1) were screened in button mushroom samples, including benzyl alcohol, 3-octanol, 3-octanone, 1-octen-3-one, isovaleraldehyde and furfural.

**Figure 3:**
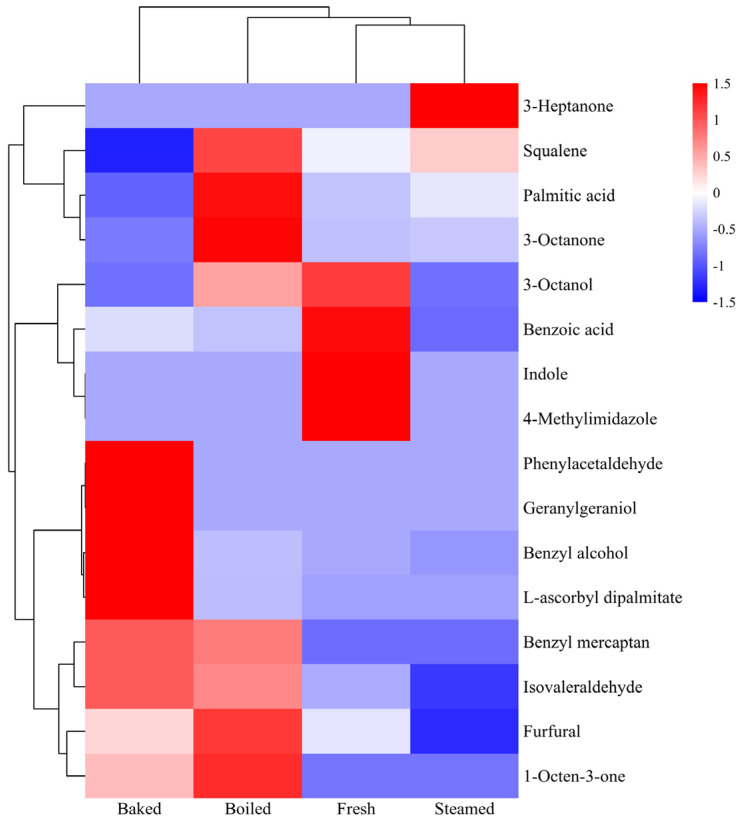
Clustering heat map of differential aroma components.

To analyze the cooking-induced flavor differences more intuitively, a clustering heat map analysis was performed using the relative contents of the 16 differential aromas, and the results are shown in Figure 3. The figure reveals that clustering initially occurred between the raw and steamed samples at the minimum distance level, aligning with the OPLS-DA analysis plot of aroma components. This suggested a higher similarity in volatiles between the raw and steamed groups. As the Euclidean distance increased, the steamed and boiled groups gradually clustered together, indicating some similarities between these two clusters. Ultimately, the roasted samples were grouped with the other samples. However, the red part of the figure highlights differences in characteristic aroma substances among the four sample groups. These variations could be attributed to the increase in heterocyclic compounds during cooking, the initial loss of volatile compounds and the thermal degradation reactions of amino acids and fatty acids [37].

### 3.4. OAV Analysis

After OPLS-DA modeling and clustering heat map analysis, we found that there were significant differences in the aroma characteristics of button mushroom from various cooking methods. However, the level of aroma components alone cannot determine their contribution to the overall aroma because the contribution of volatiles to the overall aroma depends not only on their concentration but also on their odor threshold [56]. For this reason, the degree of volatiles’ contribution can be measured by the OAV (see Table 3 for details). The investigation into thresholds revealed that 29 out of 73 aroma compounds of button mushroom lacked odor thresholds and were consequently not listed in Table 3. However, the 44 screened aroma compounds could be pivotal in evaluating the impact of various cooking methods on the flavor profile of button mushroom. Table 3 presents the published thresholds for aroma compounds in water, since water was the main medium in this study. Usually, substances with OAVs ranging from 0.1 to 1 act as flavor modifiers and have less impact on the overall aroma, while substances with OAVs greater than 1 are most likely to be the primary contributors to the overall aroma [46]. According to Table 3, we found that 15, 7, 15 and 13 volatiles had OAVs greater than 1 in raw, steamed, boiled and roasted samples, respectively. Among these volatiles, 1-octen-3-one exhibited the highest OAV, aligning with the findings of Zhang et al. [57] regarding the key aroma compounds of Boletus edulis, where 1-octen-3-one also had the highest OAV. Consequently, we concluded that 1-octen-3-one was the primary contributing substance to the overall aroma, followed by phenylacetaldehyde and methyl palmitate, which may have contributed to the aroma of button mushroom.

In summary, the different treatments (raw, steamed, boiled and roasted) affected the OAVs of volatiles. 1-Octen-3-one may be the main contributor to the aroma of button mushroom, while other volatiles such as phenylacetaldehyde and methyl palmitate also played a role in the formation of the aroma. These findings provide valuable insights for a better understanding and utilization of the flavor characteristics of button mushroom.

### 3.5. Aroma Recombination and Omission Test

**Figure 4:**
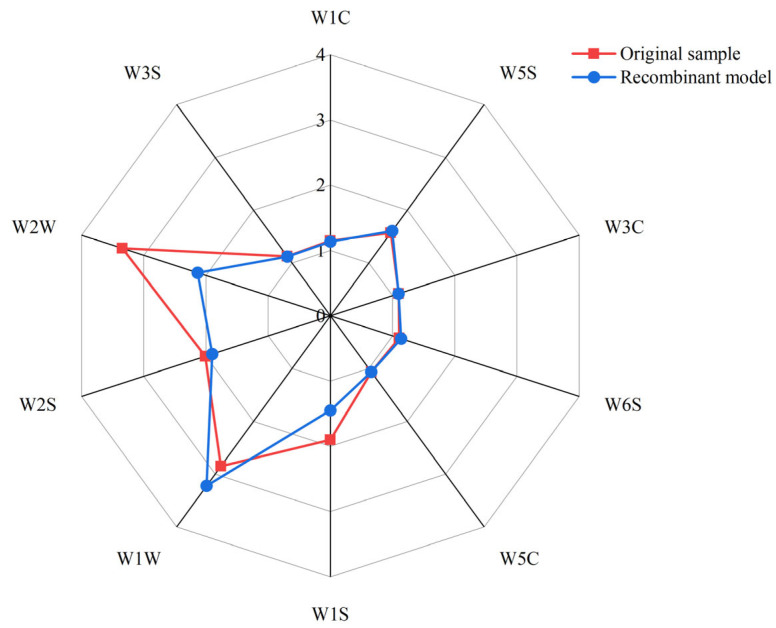
Aroma profiles of original sample and recombination model.

After the comprehensive analysis, the following conclusion can be drawn: the roasted group was the richest in flavor substances and presented the most distinct flavor profile in the E-nose radargram. Subsequently, aroma reorganization and omission experiments were carried out to further identify the key aroma components of the roasted samples. To assess the contribution and significance of aroma compounds with OAVs exceeding 1 to the overall flavor in the roasted group, we performed aroma reconstitution and omission tests based on standard aroma substances and ultrapure water. As depicted in Figure 4, the overall aroma of the reconstitution experiments had good similarity with the original samples, indicating that the preparation of the reconstitution model was successful. Building upon the successful aroma reconstitution, we also used omission tests to verify the contribution of 13 compounds with OAV ≥ 1 to the overall aroma. If the omission model after the removal of a single compound showed no significant difference from the fully reconstituted samples, it suggests that the removed compound contributed less to the overall aroma; conversely, it indicates a greater contribution to the overall aroma [58]. The results are shown in Table 4. The omission of 1-octen-3-one, benzyl alcohol, methyl valerate, 1-octen-3-ol, isovaleraldehyde, furfuryl mercaptan and heptanal exhibited significant differences in the E-nose response value compared with the recombination sample. These compounds that significantly affected the overall aroma of the reconstituted samples were considered to be the most crucial aroma substances, essential for the aroma quality of roasted button mushroom.

## 4. Conclusions

The experiment was conducted to study the aroma components of button mushroom under different cooking methods, and a total of 73 aroma components were detected. The present study revealed significant differences in the aroma components of button mushroom under various cooking methods, exhibiting unique culinary characteristics. In particular, the most intense and attractive aroma was produced by the grilled treatment. This research identified 16 differential aroma substances related to the aroma quality of button mushroom for the first time, based on the *p*-value and VIP value. In addition, OAV analysis suggested that 1-octen-3-one, phenylacetaldehyde and methyl palmitate could be the primary contributors to the aroma of roasted button mushroom. The seven key aromas of the roasted samples identified through OAV analysis and omission experiments were 1-octen-3-one, benzyl alcohol, methyl valerate, 1-octen-3-ol, isovaleraldehyde, furfuryl mercaptan and heptanal. The study revealed several volatiles with OAVs exceeding 1, notably 1-octen-3-one with OAVs ranging from 49,600 to 84,200, indicating its significance as a key aroma active compound. These findings offer valuable insights into the aroma composition and potential reactions of cooked button mushroom, which is important for culinary and food industry applications.

Given the limitations of GC-MS technology, further combination with other analytical techniques such as GC-O (gas chromatography-olfactometry) and GC-IMS (gas chromatography-ion mobility spectrometry) is necessary to enhance the study. Additionally, the pathways of flavor substance generation in button mushroom during cooking were explored to establish an objective basis for the high-quality cooking of button mushroom.

## Figures and Tables

**Table 1:** Qualitative and quantitative analysis of volatile compounds in raw and differently cooked button mushroom.

No.	Volatile Compounds	CAS	RT	Odorant	Concentration (μg/g)				*p*-Value	VIP
			(Min)	Description	Fresh	Steamed	Boiled	Baked		
Alcohols										
1	**Benzyl alcohol**	100-51-6	24.097	floral	4.55 ± 0.12 ^c^	2.32 ± 0.20 ^d^	7.26 ± 0.20 ^b^	47.56 ± 1.50 ^a^	4.01 × 10^−12^	3.24
2	2,3-Butanediol	513-85-9	18.286	-	0.30 ± 0.02	ND	ND	ND	2.33 × 10^−10^	0.37
3	1,3-Butanediol	107-88-0	18.527	-	1.01 ± 0.09	ND	ND	ND	4.38 × 10^−9^	0.67
4	3-Methyl-1-butanol	123-51-3	24.711	alcoholic, fruity, fatty, banana-like	0.31 ± 0.02	ND	ND	ND	5.31 × 10^−10^	0.37
5	Isosorbide	652-67-5	22.972	-	ND	ND	ND	0.33 ± 0.03	2.26 × 10^−9^	0.29
6	Xylitol	87-99-0	20.564	-	ND	ND	ND	0.54 ± 0.09	7.25 × 10^−7^	0.37
7	1-Octen-3-ol	3391-86-4	21.946	mushroom-like	ND	ND	1.66 ± 0.17 ^a^	0.42 ± 0.03 ^b^	5.03 × 10^−8^	0.72
8	**Geranylgeraniol**	24034-73-9	33.572	-	ND	ND	ND	5.96 ± 0.13	8.98 × 10^−14^	1.22
9	**3-Octanol**	589-98-0	22.481	earthy, mushroom-like	2.07 ± 0.12 ^a^	ND	1.45 ± 0.20 ^b^	ND	3.17 × 10^−8^	1.02
10	1-Butanol	71-36-3	20.236	floral, fragrant, fruity	ND	ND	0.79 ± 0.04	ND	4.75 × 10^−9^	0.52
11	1-Nonanol	143-08-8	22.671	green, fatty, sweet	0.63 ± 0.06	ND	ND	ND	8.74 × 10^-9^	0.53
12	1-Tetradecanol	112-72-1	30.917	waxy	0.25 ± 0.09	ND	ND	ND	4.19 × 10^−4^	0.32
Esters										
13	Methyl palmitate	112-39-0	29.961	-	0.31 ± 0.10 ^b^	ND	0.49 ± 0.12 ^a^	0.19 ± 0.07 ^b^	1.00 × 10^−3^	0.42
14	Dibutyl phthalate	84-74-2	29.412	-	0.60 ± 0.06	ND	ND	ND	8.65 × 10^−9^	0.52
15	4-Hydroxybutyl acrylate	2478-10-6	28.818	-	ND	ND	ND	1.85 ± 0.07	6.62 × 10^−12^	0.68
16	Ethyl acetate	141-78-6	19.33	fruity, sweet	0.93 ± 0.07 ^a^	0.46 ± 0.11 ^b^	0.44 ± 0.08 ^b^	0.31 ± 0.05 ^b^	6.10 × 10^−5^	0.45
17	Methyl linoleate	112-63-0	37.345	-	0.75 ± 0.05 ^a^	ND	ND	0.40 ± 0.05 ^b^	1.26 × 10^−8^	0.59
18	Methyl valerate	624-24-8	22.158	-	ND	ND	ND	0.28 ± 0.08	5.60 × 10^−5^	0.26
19	Heptadecanoic acid, heptadecyl ester	36617-50-2	27.682	-	ND	ND	2.44 ± 0.08 ^a^	0.62 ± 0.12 ^b^	3.07 × 10^−10^	0.88
20	1,3-Dipalmitin	502-52-3	25.549	-	ND	ND	ND	0.91 ± 0.05	2.47 × 10^−10^	0.48
21	**L-ascorbyl dipalmitate**	28474-90-0	26.106	-	ND	ND	1.47 ± 0.15 ^b^	17.82 ± 0.51 ^a^	1.21 × 10^−12^	2.06
22	Glycerol trioctanoate	538-23-8	25.363	-	0.69 ± 0.08 ^b^	0.42 ± 0.09 ^c^	0.93 ± 0.03 ^a^	ND	4.77 × 10^−7^	0.51
23	Ethyl 2-chlorobenzoate	7335-25-3	23.582	-	ND	0.06 ± 0.01	ND	ND	8.01 × 10^−8^	0.17
24	Ethyl isovalerate	108-64-5	22.814	fruity, sweet, grape-like	ND	0.49 ± 0.07	ND	ND	3.89 × 10^−7^	0.50
25	bis(2-ethylhexyl) phthalate	117-81-7	25.117	-	ND	1.98 ± 0.10 ^b^	2.82 ± 0.12 ^a^	2.74 ± 0.07 ^a^	5.58 × 10^−10^	0.96
26	Methyl stearate	112-61-8	24.522	-	ND	ND	0.36 ± 0.08	ND	7.00 × 10^−6^	0.35
27	Diethylene glycol monostearate	106-11-6	24.346	-	0.53 ± 0.09	ND	ND	ND	9.45 × 10^−7^	0.49
28	2-Hydroxy-n-butyric acid ethyl ester	52089-54-0	21.094	-	1.87 ± 0.05	ND	ND	ND	3.17 × 10^−13^	0.91
29	Diisobutyl phthalate	84-69-5	31.856	-	0.24 ± 0.05	ND	ND	ND	9.00 × 10^−6^	0.32
30	Dimethyl sulfite	616-42-2	14.059	-	0.17 ± 0.02	ND	ND	ND	2.67 × 10^−7^	0.27
31	Ethyl benzoate	93-89-0	20.911	-	0.19 ± 0.07	ND	ND	ND	4.31 × 10^−4^	0.28
32	Methyl myristate	124-10-7	28.569	-	0.20 ± 0.02	ND	ND	ND	2.32 × 10^−8^	0.30
33	Benzaldehyde	100-52-7	17.227	almond-like	0.29 ± 0.09 ^c^	0.22 ± 0.02 ^c^	0.45 ± 0.09 ^b^	2.80 ± 0.05 ^a^	1.50 × 10^−10^	0.78
34	**Phenylacetaldehyde**	122-78-1	17.785	honey-like	ND	ND	ND	14.53 ± 0.31	7.42 × 10^−14^	1.90
35	Hexanal	66-25-1	23.865	green	ND	ND	ND	0.30 ± 0.04	5.87 × 10^−8^	0.27
36	(2E)-2-Octadecenal	51534-37-3	33.088	-	ND	ND	2.71 ± 0.03	ND	1.11 × 10^−15^	0.97
37	2-Carboxybenzaldehyde	119-67-5	31.285	-	0.17 ± 0.03	ND	ND	ND	1.00 × 10^−6^	0.27
38	**Furfural**	98-01-1	21.348	bread-like, sweet, fatty, almond-like	1.98 ± 0.12 ^c^	0.26 ± 0.07 ^d^	4.06 ± 0.20 ^a^	2.59 ± 0.11 ^b^	4.55 × 10^−9^	1.12
39	**Isovaleraldehyde**	590-86-3	23.368	apple-like, malty	7.34 ± 0.23 ^c^	3.27 ± 0.12 ^d^	14.33 ± 0.26 ^b^	15.92 ± 0.14 ^a^	1.93 × 10^−12^	1.88
40	Decanal	112-31-2	32.153	floral, green, fruity, waxy, orange-like,	ND	ND	0.45 ± 0.10 ^b^	1.89 ± 0.06 ^a^	3.61 × 10^−10^	0.65
41	oct-2-enal	2363-89-5	29.133	fruity, green, nutty	ND	ND	2.17 ± 0.09 ^b^	2.87 ± 0.06 ^a^	5.93 × 10^−12^	0.85
42	Heptanal	111-71-7	27.4	green, fatty	0.29 ± 0.06 ^d^	1.15 ± 0.13 ^c^	3.42 ± 0.07 ^b^	3.85 ± 0.07 ^a^	4.54 × 10^−11^	0.94
43	Vanillin	121-33-5	28.166	-	0.41 ± 0.02 ^a^	ND	0.33 ± 0.02 ^b^	ND	3.11 × 10^−10^	0.46
44	Octanal	124-13-0	29.744	citrus-like, fatty	0.25 ± 0.04 ^b^	0.22 ± 0.03 ^b^	0.30 ± 0.02 ^a^	ND	2.00 × 10^−6^	0.27
Acids										
45	Acetic acid	64-19-7	16.007	sour	ND	1.42 ± 0.04 ^a^	0.72 ± 0.11 ^b^	0.69 ± 0.07 ^b^	5.57 × 10^−8^	0.82
46	**Benzoic acid**	65-85-0	20.728	bitter gourd-like	6.66 ± 0.21 ^a^	0.89 ± 0.07 ^d^	2.18 ± 0.14 ^c^	2.56 ± 0.13 ^b^	1.92 × 10^−10^	1.63
47	Lauric acid	143-07-7	30.203	tallow-like	0.79 ± 0.06 ^b^	0.55 ± 0.08 ^c^	1.09 ± 0.16 ^a^	0.99 ± 0.16 a ^b^	3.00 × 10^−3^	0.40
48	**Palmitic acid**	57-10-3	34.048	-	4.64 ± 0.12 ^c^	6.26 ± 0.09 ^b^	18.12 ± 0.16 ^a^	0.36 ± 0.11 ^d^	7.22 × 10^−15^	2.25
49	Heptanoic acid	111-14-8	26.343	-	ND	ND	ND	0.25 ± 0.11	1.00 × 10^−3^	0.24
Ketones										
50	Methylheptenone	110-93-0	23.159	sweet, fruity	ND	ND	0.58 ± 0.11	ND	2.00 × 10^−6^	0.45
51	**3-Octanone**	106-68-3	11.184	earthy, mushroom-like, resinous	0.96 ± 0.17 ^b^	1.08 ± 0.14 ^b^	5.25 ± 0.22 ^a^	ND	6.32 × 10^−10^	1.26
52	**3-Heptanone**	106-35-4	11.013	fruity	ND	1.97 ± 0.17	ND	ND	5.26 × 10^−9^	1.00
53	Geranylacetone	3796-70-1	12.536	green, fruity	0.20 ± 0.06	ND	ND	ND	3.70 × 10^−5^	0.30
54	2-Undecanone	112-12-9	12.267	floral	0.31 ± 0.04	ND	ND	ND	4.51 × 10^−8^	0.37
55	Carvone	99-49-0	12.043	-	1.34 ± 0.13 ^a^	1.23 ± 0.14 ^a^	ND	ND	1.07 × 10^−7^	0.62
56	**1-Octen-3-one**	4312-99-6	11.641	metallic, mushroom-like	ND	ND	4.21 ± 0.14 ^a^	2.48 ± 0.24 ^b^	5.60 × 10^−10^	1.12
57	2-Nonanone	821-55-6	11.835	fruity, green, cheese-like	ND	ND	ND	0.08 ± 0.01	1.30 × 10^−8^	0.14
58	Acetophenone	98-86-2	11.329	hawthorn-like	ND	ND	0.53 ± 0.20	ND	3.26 × 10^−4^	0.42
Others										
59	Undecane	1120-21-4	15.804	-	0.90 ± 0.09	ND	ND	ND	9.88 × 10^−9^	0.63
60	**Squalene**	111-02-4	36.81	-	3.29 ± 0.26 ^c^	4.32 ± 0.15 ^b^	6.49 ± 0.22 ^a^	ND	7.04 × 10^−10^	1.27
61	2,6-Dimethoxyphenol	91-10-1	33.76	clove-like, smoky	ND	0.26 ± 0.11	ND	ND	1.00 × 10^−3^	0.35
62	Isoeugenol	97-54-1	34.525	clove-like	ND	0.43 ± 0.12	ND	ND	4.70 × 10^−5^	0.46
63	**4-Methylimidazole**	822-36-6	16.783	-	2.37 ± 0.20	ND	ND	ND	4.45 × 10^−9^	1.03
64	**Indole**	120-72-9	33.252	musty	11.51 ± 0.26	ND	ND	ND	1.00 × 10^−13^	2.26
65	2-Butoxyethanol	111-76-2	18.824	-	0.20 ± 0.08	ND	ND	ND	1.00 × 10^−3^	0.29
66	2-Methylpyrazine	109-08-0	31.076	nutty, sweet	0.21 ± 0.12 ^a^	ND	ND	0.27 ± 0.07 ^a^	2.00 × 10^−3^	0.32
67	4-Isopropylphenol	99-89-8	31.623	-	ND	0.24 ± 0.07 ^b^	0.31 ± 0.03 ^a^	ND	9.00 × 10^−6^	0.35
68	Butylated Hydroxytoluene	128-37-0	34.209	-	ND	ND	0.55 ± 0.07	ND	7.17 × 10^−8^	0.43
69	1-ethoxyethylbenzene	3299-05-6	35.403	-	ND	ND	ND	0.32 ± 0.04	8.24 × 10^−8^	0.28
70	Cycloheptatriene	544-25-2	13.493	-	ND	ND	ND	0.40 ± 0.02	3.56 × 10^−10^	0.31
71	**Benzyl mercaptan**	100-53-8	37.082	-	ND	ND	40.51 ± 1.19 ^b^	45.38 ± 0.46 ^a^	2.88 × 10^−13^	3.57
72	2-Butylfuran	4466-24-4	33.938	noncharacteristic	ND	0.60 ± 0.06 ^a^	0.39 ± 0.02 ^b^	0.44 ± 0.02 ^b^	2.23 × 10^−7^	0.52
73	Furfuryl mercaptan	98-02-2	25.795	-	ND	ND	ND	0.24 ± 0.12	3.00 × 10^−3^	0.23

Each value is expressed as the mean ± SD (*n* = 3) of triplicate determinations. Significant differences between samples from different cooking methods were analyzed using ANOVA and Duncan’s test, and specific *p*-values are presented in the table. We employed supervised regression modeling using OPLS-DA to obtain variable importance in projection (VIP). Means with different letters within a row are significantly different (*p* < 0.05) in different cooking methods. ND means that the compounds were not detected. RT: Retention time. The use of “-” indicates that the odorant description of the compounds was not found. Differential aroma components are shown in bold.

**Table 2:** Classification and content of volatile substances.

Type of Compounds	Fresh	Contents (µg/g)	Steamed	Contents (µg/g)	Boiled	Contents (µg/g)	Baked	Contents (µg/g)
Alcohols	7	9.11	1	2.32	4	11.16	5	54.81
Esters	11	6.48	5	3.41	7	8.94	9	25.13
Aldehydes	7	10.73	5	5.12	9	28.22	8	44.76
Acids	3	12.09	4	9.12	4	22.12	5	4.86
Ketones	4	2.82	3	4.28	4	10.58	2	2.56
Others	5	18.49	5	5.84	5	48.24	6	47.04
Total	37	59.72	23	30.09	33	129.26	35	179.16

The aroma content of each sample is shown as a mean value.

**Table 3:** Changes in OAV of aroma compounds in button mushroom during cooking.

No.	Compounds	Odor Threshold µg/g	OAV			
			Fresh	Steamed	Boiled	Baked
1	Benzyl alcohol	2.55	1.78	0.91	2.85	18.65
2	2,3-Butanediol	0.0951	3.18	ND	ND	ND
3	1,3-Butanediol	10	0.10	ND	ND	ND
4	3-Methyl-1-butanol	0.25	1.24	ND	ND	ND
5	1-Octen-3-ol	0.1	ND	ND	16.60	4.17
6	3-Octanol	0.042	49.26	ND	34.55	ND
7	1-Butanol	0.5	ND	ND	1.58	ND
8	1-Nonanol	0.09	7.04	ND	ND	ND
9	1-Tetradecanol	559	0.00	ND	ND	ND
10	Methyl palmitate	0.003	103.67	ND	163.33	62.67
11	Ethyl acetate	5	0.19	0.09	0.09	0.06
12	Methyl valerate	0.02	ND	ND	ND	14.00
13	2-Hydroxy-n-butyric acid ethyl ester	0.8	2.34	ND	ND	ND
14	Ethyl benzoate	0.05	3.80	ND	ND	ND
15	Benzaldehyde	0.35	0.83	0.63	1.29	8.00
16	Phenylacetaldehyde	0.004	ND	ND	ND	3632.50
17	Hexanal	0.02	ND	ND	ND	15.00
18	Furfural	9.56	0.21	0.03	0.42	0.27
19	Isovaleraldehyde	13	0.56	0.25	1.10	1.22
20	Decanal	33	ND	ND	0.01	0.06
21	oct-2-enal	394	ND	ND	0.01	0.01
22	Heptanal	0.06	4.83	19.17	57.00	64.17
23	Octanal	0.008	31.25	27.50	37.50	ND
24	Acetic acid	22	ND	0.06	0.03	0.03
25	Benzoic acid	1	6.66	0.89	2.18	2.56
26	Lauric acid	59	0.01	0.01	0.02	0.02
27	Palmitic acid	503	0.01	0.01	0.04	0.00
28	Heptanoic acid	10.4	ND	ND	ND	0.02
29	Methylheptenone	0.85	ND	ND	0.68	ND
30	3-Octanone	0.07	13.71	15.43	75.00	ND
31	3-Heptanone	0.08	ND	24.63	ND	ND
32	Geranylacetone	0.06	3.33	ND	ND	ND
33	2-Undecanone	0.182	1.70	ND	ND	ND
34	Carvone	0.1	13.40	12.30	ND	ND
35	1-Octen-3-one	0.00005	ND	ND	84,200.00	49,600.00
36	2-Nonanone	0.2	ND	ND	ND	0.40
37	Acetophenone	0.065	ND	ND	8.15	ND
38	Undecane	10	0.09	ND	ND	ND
39	2,6-Dimethoxyphenol	1.85	ND	0.14	ND	ND
40	Isoeugenol	0.1	ND	4.30	ND	ND
41	Indole	25	0.46	ND	ND	ND
42	Butylated Hydroxytoluene	0.161	ND	ND	3.42	ND
43	2-Butylfuran	0.005	ND	120.00	78.00	88.00
44	Furfuryl mercaptan	0.01	ND	ND	ND	23.80

Odor thresholds for volatile compounds were obtained from http://www.odour.org.uk (accessed on 21 February 2024) and the *Compilations of Odour Threshold Values in Air, Water and Other Media*. “ND” means not detected and therefore not analyzed in the OAV calculation.

**Table 4:** The results of the omission tests for the recombination model.

Compound(s) Omitted	Degree of Odor Difference
1-Octen-3-one	W2S↓ *
Benzyl alcohol	W2S↓ **
Methyl valerate	W2S↓ ***, W2W↓ ***
Phenylacetaldehyde	ns
1-Octen-3-ol	W2S↓ ***, W2W↓ ***
Benzoic acid	ns
Isovaleraldehyde	W2S↓ ***
Hexanal	ns
Methyl palmitate	ns
Furfuryl mercaptan	W2S↓ ***, W2W↓ ***
2-Butylfuran	ns
Heptanal	W2S↓ ***
Benzaldehyde	ns

ns, not significant; *, *p* < 0.05; **, *p* < 0.01; ***, *p* < 0.001. ↓, decrease in odor response values.

## Data Availability

The original contributions presented in the study are included in the article, further inquiries can be directed to the corresponding author.
